# IGRF-14 secular variation prediction from core surface flow acceleration

**DOI:** 10.1186/s40623-025-02347-x

**Published:** 2026-01-16

**Authors:** Frederik Dahl Madsen, Ciarán D. Beggan, William J. Brown, Richard Holme, Jonas Bregnhøj Lauridsen, Kathryn A. Whaler

**Affiliations:** 1 School of GeoSciences, University of Edinburgh, Edinburgh, UK; 2 British Geological Survey, Lyell Centre, Edinburgh, UK; 3 School of Environmental Sciences, University of Liverpool, Liverpool, UK; 4 DTU Space, Technical University of Denmark, Kongens Lyngby, Denmark

**Keywords:** Geomagnetism, Geomagnetic jerks, Core surface flow modelling, Satellite magnetism, IGRF-14

## Abstract

**Abstract:**

The International Geomagnetic Reference Field (IGRF) has been regularly updated since its inception in 1965. Every recent iteration contains an estimate of the geomagnetic secular variation (SV), for the intermediate years between iterations. We submit a candidate model for the geomagnetic secular variation (SV) for the period 2025–2030 for the 14^th^ generation of the IGRF. Given the recent evidence in the geomagnetic SV record for core surface waves, we forecast SV based on the periodic behaviour of core surface flow acceleration. We obtain an advective core surface flow model, in terms of poloidal and toroidal flow coefficients, from spatial gradients of SV geomagnetic virtual observatory data from the low-Earth orbiting CHAMP and Swarm missions from January 2001 to January 2010, and April 2014 to January 2024, respectively. From these, we calculate the flow acceleration coefficients from the first time-derivative. This assumes the flow is spatio-temporally simple, without imposing any physical constraints on its geometry. We fit each acceleration coefficient with a sinusoidal function, which is used to extrapolate 6 years into the future. These sinusoidally varying acceleration time series are integrated over time to obtain the core flow coefficients, which are then used to predict the average advected SV over the 5-year IGRF period. We recreate previous IGRF predictions using our CHAMP-based flows to validate our methodology, which we find to outperform previous IGRF iterations, and use the Swarm-based flows to forecast the SV for IGRF-14. Our Swarm-based model predicts sudden changes in SV—also known as geomagnetic jerks—in 2024 in the Equatorial Pacific, and in 2028 in the region around central Africa. Although the IGRF SV is a snapshot over a 5-year period, allowing for periodic behaviour offers potential improvements over other methods of prediction.

**Graphical Abstract:**

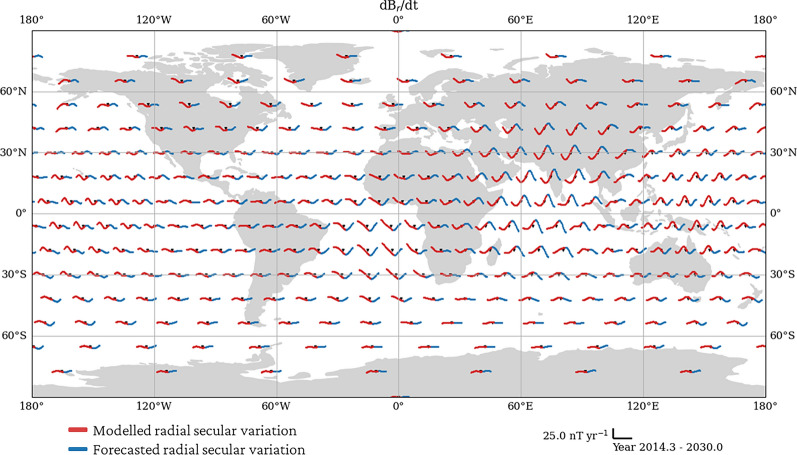

**Supplementary Information:**

The online version contains supplementary material available at 10.1186/s40623-025-02347-x.

## Introduction

The International Geomagnetic Reference Field (IGRF) has been regularly updated since its first iteration in 1965 (Zmuda 1971), and is an important, collaborative output from the global geomagnetic community (e.g., Alken et al. 2021a). Every iteration contains an estimate of the geomagnetic field at 5-year intervals since 1900.0, represented by values of spherical harmonic coefficients, also referred to as Gauss coefficients. As well as the Gauss coefficients, each iteration of the IGRF also contains a forecast of the geomagnetic secular variation (SV), over the subsequent 5 years, assuming the magnetic field changes linearly.

Although SV is not linear, with the IGRF it is approximated as such over short periods up to 5 years. In the past three decades, many methods for forecasting the SV for IGRF have been investigated, including linear extrapolation from the observed change in the previous year to 5 years (e.g., Alken et al. 2015; Finlay et al. 2015), using core flow velocity (e.g., Beggan and Whaler 2010), data assimilation with a geodynamo model (e.g., Sanchez et al. 2020), fitting polynomials (e.g., Petrov and Bondar 2021), and integration of stochastic equations (e.g., Baerenzung et al. 2020; Huder et al. 2020). The IGRF analysis process combines the candidate models together in a manner agreed by a voluntary taskforce (e.g., Alken et al. 2021b). Thus the final forecast model is a weighted combination of the submitted candidates, each of which has a different approach to predicting SV.

**Fig. 1:**
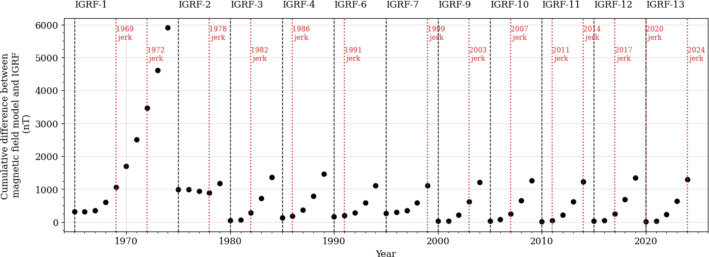
Cumulative absolute difference between Gauss coefficients from IGRF, using the SV forecast from each IGRF generation, and geomagnetic field models COV–OBS.x2 (1965–2020, Huder et al. 2020) or CHAOS 7.18 (2020–2025, Finlay et al. 2020), up to degree and order 8. Timings of geomagnetic jerks are highlighted in red. Jerk dates are compiled from Brown et al. (2013); Mandea et al. (2010); Holme and de Viron (2013); Chulliat and Maus (2014); Kloss and Finlay (2019); Pavón-Carrasco et al. (2021); Whaler et al. (2022); Madsen et al. (2025)

Figure 1 shows the cumulative absolute difference between the predicted Gauss coefficients for each IGRF model (Zmuda 1971; IAGA Division I Study Group on Geomagnetic Reference Fields 1975; Peddie 1982; Barraclough 1987; Langel 1992; Barton 1997; International Association of Geomagnetism and Aeronomy (IAGA) 2003, 2005; Finlay et al. 2010; Thébault et al. 2015; Alken et al. 2021a) and that of observation-based continuous models, such as COV–OBS.x2 (1965–2020, Huder et al. 2020) and CHAOS 7.18 (2020–2025, Finlay et al. 2020). It shows that the accuracy of the IGRF predictions worsen within a few years of release, and in general the forecast skill has not improved much since 1980. This is because SV is not constant with time. There are, furthermore, regions where spatiotemporally localised acceleration of the field occurs (e.g., Chulliat and Maus 2014). One manifestation of this non-linearity is the effect of geomagnetic jerks – defined as inflections in SV (e.g., Courtillot et al. 1978), likely triggered by hydromagnetic waves propagating within the outer core (e.g., Aubert and Finlay 2019; Finlay et al. 2023), as identified from magnetic models at the core surface (Gillet et al. 2022, 2024; Ropp and Lesur 2023; Madsen et al. 2025; Grüne et al. 2025). The times of several well-recognised geomagnetic jerks since 1965 are also plotted in Fig. 1. Thus, dynamic models are required to improve the forecast of the geomagnetic field by taking into account its more rapid changes.

From the magnetic induction equation, SV is driven by flow advection and magnetic diffusion, where the main driver for intra-decadal variation in SV is often attributed to advective flow at the surface of the Earth’s outer core (e.g., Holme 2015). However, if we neglect the effect of diffusion (also referred to as the ‘frozen flux hypothesis’ by Roberts and Scott 1965), we can relate observations of SV to core surface flow. Inverting the induction equation for core surface flow is inherently non-unique, as first noted by Roberts and Scott (1965) and later formalised by Backus (1968). Many studies utilise recent geodynamo simulations to reduce this non-uniqueness. Some authors utilise the full geodynamo with data-assimilation techniques (e.g., Sanchez et al. 2020; Fournier et al. 2021; Aubert 2023), whereas others utilise the statistical behaviour of the geodynamo to constrain stochastic elements of their flows (e.g., Huder et al. 2019; Gillet et al. 2022; Ropp and Lesur 2023; Istas et al. 2023; Gillet et al. 2024; Suttie et al. 2025). Other studies impose geometrical constraints as *a priori* information, such as tangential geostrophy or quasigeostrophy, to reduce the solution space, where the latter enforces equatorial symmetry (e.g., Pais and Jault 2008; Gillet et al. 2015; Amit and Pais 2013; Li et al. 2025). However, Rogers et al. (2025) found that the slowly varying part of inverted flow models derived using dynamo priors are sensitive to the choice of dynamo. Furthermore, there is increasing evidence for some level of equatorial asymmetry to explain the observed SV (e.g., Amit and Pais 2013; Barrois et al. 2017; Bärenzung et al. 2018; Madsen et al. 2025). When solving for spatiotemporally simple but geometrically unconstrained flows, it is found that at least 10% equatorial asymmetry is required to fit the observed SV. Thus, care needs to be taken when making core surface flow modelling choices, as the *a priori* assumptions significantly affect the flow dynamics of the model output.

Recently, quasi-periodic variations in core surface flow have been suggested to dominate intra-decadal changes in SV (e.g., Ropp and Lesur 2023; Li et al. 2024; Gillet et al. 2022, 2024; Grüne et al. 2025). We, therefore, attempt to incorporate this new discovery into a scheme to forecast SV using our estimates of the periodic variation in core surface flow acceleration. We use recent core surface flow models developed by Madsen et al. (2025), derived from spatial gradients SV data from the CHAMP and Swarm missions (Hammer et al. 2022). First, we evaluate the forecasting capabilities of this method by predicting SV with the CHAMP-derived flows. Then, we use the Swarm-derived flows to predict SV from 2024 to 2030.

We present the data from the CHAMP and Swarm satellite missions, spanning 2001–2010 and 2014–2024, in Sect. 2. We then summarise the methods used to derive the core surface flow models, expressed using spherical harmonic coefficients, and the integration that followed to obtain an expression for flow acceleration, in Sect. 3.1. We fit the core surface flow acceleration coefficients with a pure sinusoidal function, assuming that the core surface flow acceleration varies around zero over decadal timescales, i.e., the core surface flow will not accelerate indefinitely, and present this methodology in Sect. 3.2. We show these sinusoidal fits to the acceleration coefficients, extracted 5–6 years from the end of the flow models (2015 and 2030, for CHAMP and Swarm, respectively), in Sect. 4.1, and from these models, obtain a forecast of core surface flow in Sect. 4.2. Finally, we calculate and analyse the SV change from the forecasted flow in Sect. 4.4, compare our predictions to previous iterations of the IGRF (Finlay et al. 2010), and present our candidate model for the IGRF-14. We discuss these results in the context of the IGRF-14 in Sect. 5, and conclude in Sect. 6.

### Data

With the European Space Agency Swarm mission (Friis-Christensen et al. 2006), we are able to obtain the spatial gradient tensor of the magnetic field from across- and along-track differences of the satellite measurements, and along-track measurements with CHAMP. These data are more sensitive to the magnetic field and SV than the vector components (Kotsiaros and Olsen 2014), and increase the spatial resolution of our flow models derived from these by up to 40% (Madsen et al. 2025). We produce 4-monthly means of the six independent spatial gradient tensor components of magnetic field data at 300 geomagnetic virtual observatories (GVOs) from 2001.67 to 2010.33 for CHAMP and 2014.33 to 2024.00 for Swarm, following the method of Hammer et al. (2022). We then obtain SV from these components from annual first differences, attributing the SV value to the temporal midpoint. We derive the associated uncertainty estimates for each GVO tensor element from the variance of residuals between the GVO datum and the CHAOS 7.18 model estimate (Finlay et al. 2020) of each tensor element at the GVO location, as outlined by Hammer et al. (2022).

**Fig. 2:**
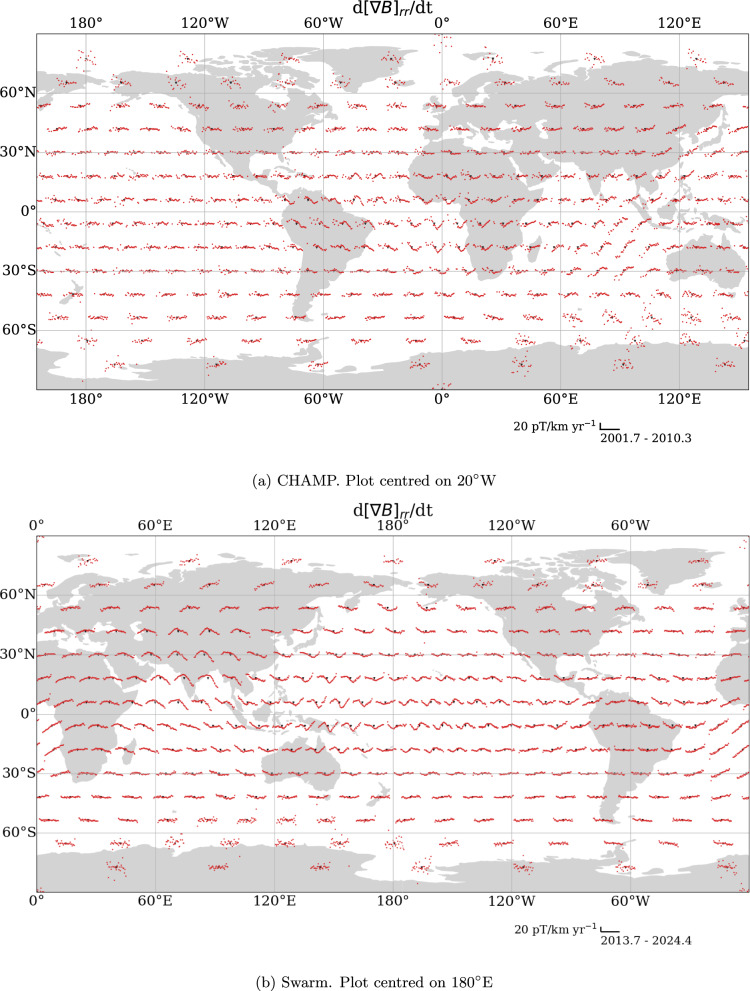
Radial derivative of radial SV (red) at each GVO (black dots) from CHAMP and Swarm. Data for each GVO are centred on the GVO location. x- and y-axes for each GVO are provided in the bottom right of the figures. The plots are in Plate Carrée projection

Figure 2 shows the GVO locations, as well as the radial gradient of the radial SV, $\dot{B}_{rr}$, at each GVO from both satellite missions. In the figure, we see V-shaped signatures in the equatorial Atlantic region in 2003 and 2007 from CHAMP, and equatorial Pacific region in 2017 and 2020 from Swarm. The latter were noted by Whaler et al. (2022) and Madsen et al. (2025) to be contemporary with the 2017 and 2020 geomagnetic jerks observed in SV vector data, although we recognise that the 2017 jerk is observed as far away as northern Asia. We, therefore, assume that the 2003 and 2007 inflections in $\dot{B}_{rr}$ in Fig. 2a are associated with the 2003.5 (Wardinski et al. 2008) and 2007 (Chulliat et al. 2010) geomagnetic jerks, although there has not been a formal analysis of how geomagnetic jerks appear in spatial gradients of SV. We note that $\dot{B}_{rr}$ shows little change at mid-to-high latitudes, and that particularly at polar latitudes is more noisy, due to higher levels of external field contamination from ionospheric fields and field-aligned currents (e.g., Xiong et al. 2020).

## Methods

### Flow and acceleration modelling

The reduced induction equation, assuming negligible diffusion, relates the core surface flow, $\mathbf {u_H}$, to the radial geomagnetic field, $B_r$, by

1 $$\begin{aligned} \dot{B_r} + \mathbf {\nabla _H} \cdot (\mathbf {u_H} B_r) = 0, \end{aligned}$$

where $\dot{B_r} = \tfrac{\partial B_r}{\partial t}$ is its time derivative, and $\mathbf {\nabla _H}=\nabla -\hat{\textbf{r}} \cdot \nabla$ only contains horizontal derivatives.

We can consider $\textbf{B}$ as the gradient of a potential field, *V*:

2 $$\begin{aligned} \textbf{B} = -\nabla V(r,\theta ,\phi ,t), \end{aligned}$$

where *r*, $\theta$, and $\phi$ are spherical polar coordinates radius, colatitude, and longitude, respectively. Representing this potential field in spherical harmonics gives

3 $$\begin{aligned} V(r,\theta ,\phi ,t) = a \sum _{l=1}^{L_B} \sum _{m=0}^l \Big ( \frac{a}{r}\Big )^{l+1}&( g_l^m(t) \cos {m\phi } + h_l^m(t) \sin {m\phi }) P_l^m(\cos {\theta }) \end{aligned}$$

where $P_l^m(\cos {\theta })$ are Schmidt quasi-normalised associated Legendre polynomials of degree and order *l* and *m*, respectively, *a* is the reference radius (6371.2 km), and *g* and *h* are the Gauss coefficients, dependent on time, *t*. Due to the static and small-scale crustal magnetic field (e.g., Cain et al. 1989), we truncate the magnetic field and SV at degree $L_B=14$.

We decompose the core surface flow, $\mathbf {u_H}$, into its toroidal and poloidal parts which, since the radial flow vanishes at the core–mantle boundary (CMB), can be expressed as

4 $$\begin{aligned} \mathbf {u_H} = \mathbf {\nabla } \times (\mathcal {T}\hat{\textbf{r}}) + \mathbf {\nabla _H}(\mathcal {S}\hat{\textbf{r}}) \end{aligned}$$

where $\mathcal {T}$ and $\mathcal {S}$ are the toroidal and poloidal scalar potentials, respectively. Like *V*, the flow potentials can be represented in terms of spherical harmonics:

5 $$\begin{aligned} \mathcal {T}(\theta ,\phi ,t)= & \sum _{l=1}^{L_u} \sum _{m=0}^l (t_l^{m\,c}(t) \cos {m\phi } + t_l^{m\,s}(t) \sin {m\phi }) P_l^m(\cos {\theta }) \nonumber \\ \mathcal {S}(\theta ,\phi ,t)= & \sum _{l=1}^{L_u} \sum _{m=0}^l (s_l^{m\,c}(t) \cos {m\phi } + s_l^{m\,s}(t) \sin {m\phi }) P_l^m(\cos {\theta }) \end{aligned}$$

By truncating the velocity fields at degree $L_u$, it is assumed that the energy of the flow is constrained within the length-scale related to $L_u$. We truncate at $L_u=14$.

Equations (1)–(5) can be manipulated to relate the Gauss coefficients to the toroidal and poloidal flow coefficients:

6 $$\begin{aligned} \dot{\textbf{g}} = \textbf{E}\textbf{t}+\textbf{G}\textbf{s}, \end{aligned}$$

where vectors $\dot{\textbf{g}}$, $\textbf{t}$, and $\textbf{s}$, respectively, contain the SV Gauss, toroidal velocity, and poloidal velocity coefficients, and matrices $\textbf{E}$ and $\textbf{G}$ depend on the main field coefficients and the Elsasser and Gaunt integrals, respectively (Gibson and Roberts 1969; Whaler 1986). We treat the main field as known and specified by the CHAOS$-$7.18 field model (Finlay et al. 2020), also truncated to $L_B=14$. We can express the spatiotemporal derivatives of Eq. (2) as

7 $$\begin{aligned} \dot{\textbf{d}} = \textbf{Y} \dot{\textbf{g}} \end{aligned}$$

where elements of ${\textbf {Y}}$ contain spherical harmonics and their derivatives, and $\dot{\textbf{d}}$ is a vector containing the spatial gradients of SV (Kotsiaros and Olsen 2014; Whaler et al. 2022). We can then link the SV data to the flow coefficients by substituting Eq. (7) into Eq. (6):

8 $$\begin{aligned} \dot{\textbf{d}} = \textbf{YEt} + \textbf{YGs} \equiv \textbf{Am} \end{aligned}$$

Here, $\textbf{A}$ is the normal equations matrix and $\textbf{m}$ is the model vector containing the toroidal and poloidal flow coefficients. We note that as we impose the large-scale assumption—i.e., that the SV is governed by flow within spherical harmonic degree $L_u$—we neglect the small-scale flow, as well as the effect of diffusion. Other studies model both large-scale advective flow, diffusion, and the small-scale flow (i.e., flow with a spatial scale smaller than that related to $L_u$), either using a statistical approach (e.g., Eymin and Hulot 2005; Pais and Jault 2008; Gillet et al. 2015; Baerenzung et al. 2016), or with data assimilation in conjunction with numerical simulations of the geodynamo (e.g., Sanchez et al. 2020; Fournier et al. 2021; Aubert 2023).

Recognising the ill-determined nature of the problem (Holme 2015), we employ a regularised least-squares solution (e.g., Whaler 1986). We invert data from the 300 GVOs at multiple epochs simultaneously, regularising the solution both temporally and spatially (Whaler et al. 2016, 2022). We choose to regularise our model temporally by minimising the acceleration between epochs. For the spatial regularisation, we choose the ‘strong norm’ (Bloxham 1988), which minimises the second spatial derivatives of the flow, averaged across the CMB, thus penalising spatial complexity:

9 $$\begin{aligned} \int _{\Omega } \big ((\mathbf {\nabla _H}^2{u_\theta })^2 + (\mathbf {\nabla _H}^2{u_\phi })^2 \big )\text {d}\Omega , \end{aligned}$$

where $(u_\theta ,u_\phi )$ are meridional and azimuthal flow components, respectively, and $\Omega$ is the CMB.

The regularized least-squares solution to Eq. (8) thus takes the form

10 $$\begin{aligned} \hat{\textbf{m}} = (\textbf{A}^T\mathbf {C_e}^{-1}\textbf{A}+\lambda _v\mathbf {C_m}^{-1}+\lambda _t\textbf{D}^T\textbf{D})^{-1}\textbf{A}^T\mathbf {C_e}^{-1}\dot{\textbf{d}} \end{aligned}$$

where $\mathbf {C_e}$ is the data covariance matrix, which consists of the variances for each gradient datum at each GVO arranged along the diagonal, and zeroes elsewhere. $\mathbf {C_m}$ is the *a priori* model covariance matrix (implementing the strong norm), $\textbf{D}$ is the temporal first differences matrix, and $\lambda _v$ and $\lambda _t$ are the spatial and temporal damping factors, respectively. More details are given by Madsen et al. (2025). The models are computed using the conjugate gradient algorithm with Jacobi preconditioning, up to degree and order $L_u=14$.

From the flow, we obtain core surface flow acceleration by taking the discrete 4-monthly time derivative of each flow coefficient as follows:

11 $$\begin{aligned} \dot{\hat{\textbf{m}}}_{t+\frac{1}{2}}= \frac{\hat{\textbf{m}}_{t+1} - \hat{\textbf{m}}_t}{\Delta t} \end{aligned}$$

where $_t$ signifies the specific subset of $\hat{\textbf{m}}$ that corresponds to a given epoch of data and $\Delta t=\frac{1}{3}$ years is the time between successive epochs.

### Forecasting—fitting sinusoidal functions to core surface flow acceleration

Given the high-quality satellite data from CHAMP and Swarm, we can now readily resolve core surface flow acceleration. Madsen et al. (2025) and Grüne et al. (2025) showed that the flow-acceleration coefficients vary around zero on intra-decadal to decadal time-scales with some periodic tendencies.

To investigate periodic signals in the acceleration coefficients, we employ a similar method to Holme and de Viron (2013) and Madsen and Holme (2025), and set up a simple inversion scheme to solve for the best fit amplitude and phase of a sinusoidal signal for each SV Gauss coefficient, using a least-squares approach. To avoid inter-annual noise dominating the periodic signature of the acceleration coefficients, we first fit each coefficient with a damped least-squares spline (e.g., Constable and Parker 1988; Madsen and Holme 2025). Then, considering each spline as a vector, $\gamma$, of length *N*, we relate $\gamma$ to a sinusoidal signal as a function of time with period $\tau$, by

12 $$\begin{aligned} \mathbf {\gamma }=\textbf{X}\textbf{p} \end{aligned}$$

where the condition matrix $\textbf{X}$ contains the sinusoidal variations as a function of time, and the model vector ${\textbf {p}}$ contains the respective amplitudes, *A* and *B*, as

$$\textbf{X}(t) = \left[ \sin {\frac{2\pi t}{\tau }} \quad \cos {\frac{2\pi t}{\tau }} \right] ; \qquad \textbf{p} = \left[ \begin{array}{c} A \\ B \end{array} \right]$$

For any given period, we can thus calculate the best fit amplitudes by finding the least-squares solution to Eq. (12), yielding the best fit model vector $\hat{\textbf{p}}$ to the data:

13 $$\begin{aligned} \hat{\textbf{p}}=(\textbf{X}^T \textbf{X})^{-1} \textbf{X}^T \mathbf {\gamma } \end{aligned}$$

This inversion is linear, but only if $\tau$ is defined. To find the best fit period, we repeat this inversion with values of $\tau$ between 1 and 20 years, in steps of 0.1 years, for each spherical harmonic coefficient of flow acceleration. Given that we have only 10 years of Swarm SV data, we allow for $\tau$ to increase up to 20 years as a proxy for flow-acceleration coefficients that may appear to vary in a strictly linear fashion over the past 10 years. We pick the best fit period, where there is a minimum in the model misfit to the coefficient. If there is no peak, we set $\tau =20$ years. If there is more than one periodic fit, we pick the peak with the higher amplitude.

After finding the best fit period for each flow-acceleration coefficient, we create a sinusoidal time-series in the period of 2001.67–2015.00 for CHAMP, and 2014.33–2030.00 for Swarm. We then integrate the best sinusoidal fit to each of the flow acceleration coefficients over the whole period, to obtain a model of flow velocity using the initial velocity from our flow model obtained from Eq. (10). We refer to the predicted flow acceleration—and associated velocity—models as our sinusoidal models. Finally, we obtain SV estimates from our sinusoidal flow model, using Eq. (6). In this step, we assume that the main field is constant in our forecasting period. To evaluate the performance of our model, we choose different timepoints to start the integration from. For the CHAMP-based models, we integrate from 2005 and 2010, to compare our predicted SV to IGRF-10 and IGRF-11, respectively. For our Swarm-based models, we similarly integrate from 2015 and 2020, to create comparison SV predictions to IGRF-12 and IGRF-13. Finally, for our IGRF-14 candidate model, we integrate from the end of our model, which is in 2024. For any given IGRF SV prediction, we calculate the temporal mean of each SV coefficient between IGRF timespan (e.g., 2010–2015 for IGRF-11), and attribute it to the temporal midpoint. These constitute our comparison models for the previous IGRF iterations, and our IGRF-14 SV candidate model.

## Results

**Fig. 3:**
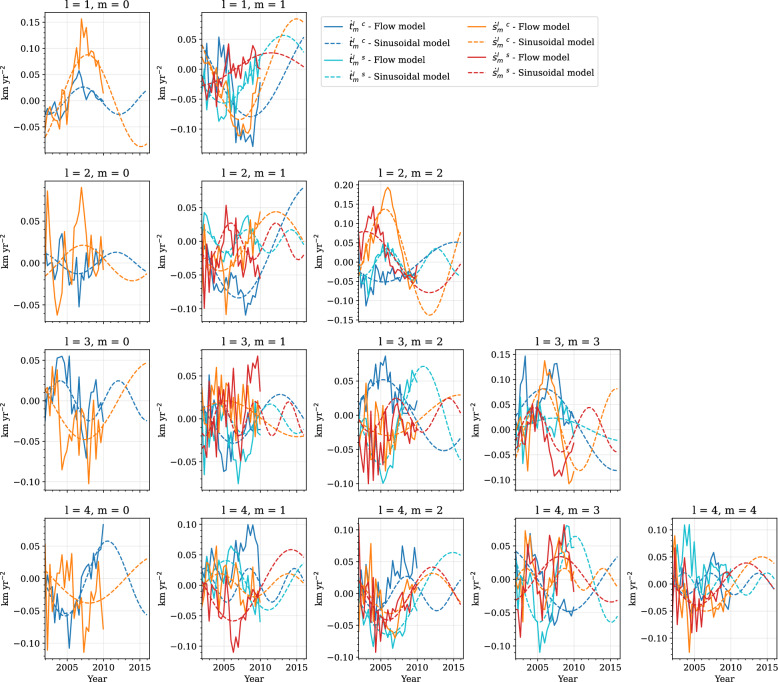
Toroidal (blue and turquoise) and poloidal (orange and red) flow-acceleration coefficients for CHAMP. Solid lines show flow acceleration from the core surface flow model, and dashed lines show the sinusoidal model. Rows show spherical harmonic degree, and columns spherical harmonic order. Note that the *y*-axis scale varies for each order and degree

**Fig. 4:**
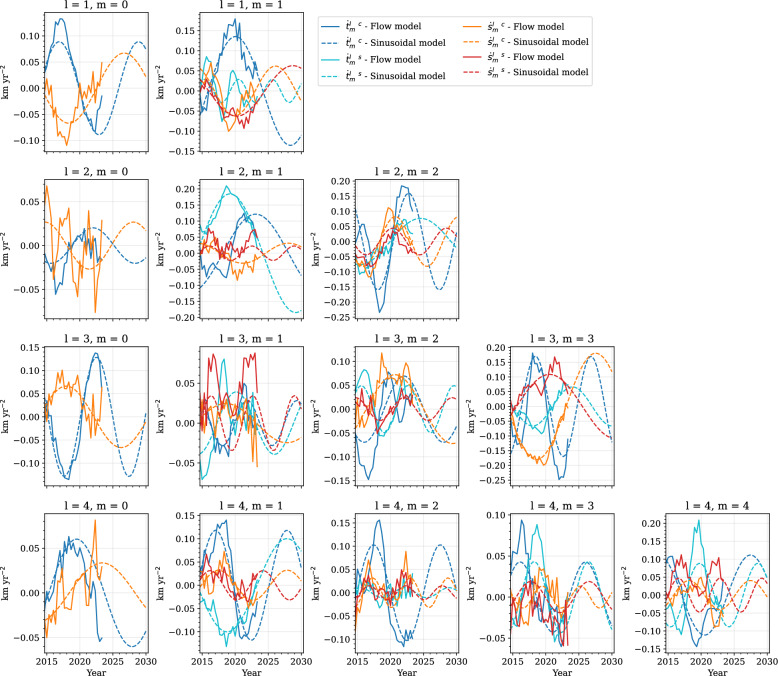
Same as Fig. 3, but for Swarm

### Fitting periodic trends to acceleration coefficients

Details of damping parameters and model resolution for the two flow models are given by Madsen et al. (2025), and are also summarised in Supplementary Table A2. We obtain the flow-acceleration coefficients from first differences, as given by Eq. (11). Figures 3 and 4 show the acceleration coefficients obtained from our core surface models in solid lines, up to degree and order 4, for CHAMP and Swarm, respectively. We see that many coefficients, for example, $\dot{t}_3^{0~c}$ and $\dot{s}_3^{0~c}$ in Fig. 4, show convincingly periodic behaviour during the Swarm era. Some coefficients, however, like $\dot{s}_4^{0~c}$ in Fig. 4, appear to behave in a more linear manner. The sinusoidal predictions are also shown in Figs. 3 and 4, extended to 2015 and 2030, respectively, as dashed lines. We see that they manage to capture the overall trend of the coefficients, where coefficients vary around zero. However, a few coefficients, such as $\dot{s}_4^{4~s}$ in Fig. 4, vary periodically at an offset from zero, and so the periodic fit is shifted with respect to the observed acceleration.

**Fig. 5:**
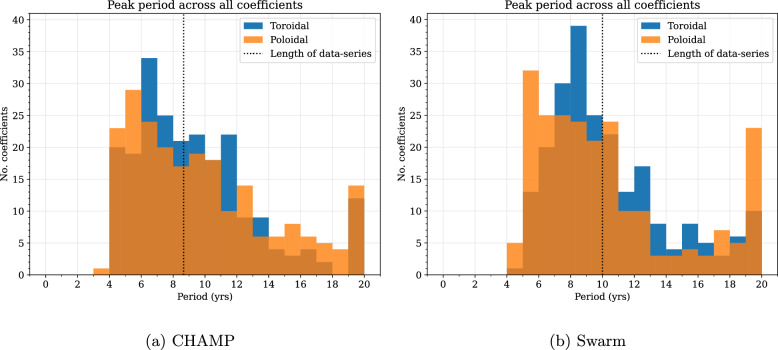
Histogram of best-fit periods to flow-acceleration coefficients up to spherical harmonic degree and order $L_u=14$ for CHAMP (**a**) and Swarm (**b**). Blue represents toroidal, and orange represents poloidal, flow coefficients. Vertical dashed lines indicate the length of the data series

Figure 5 shows the distribution of periods across all 448 acceleration coefficients up to degree and order 14 for each flow model. The best-fitting periods for most acceleration coefficients were $\le 12$ years for both models. For Swarm, the toroidal flow-acceleration coefficient period distribution peaks between 8 and 9 years, whereas the poloidal flow-acceleration coefficient periods have a flatter distribution predominantly between 5 and 11 years. For CHAMP, the distribution in best-fit periods for both the toroidal and poloidal flow-acceleration coefficients appear long-tailed, with a peak in periods of around 6 years. For Swarm, twice as many poloidal flow-acceleration coefficients did not have any strong oscillatory signals (23 out of 224, compared to 10 toroidal coefficients showing no periodic signatures), whereas for CHAMP, the divide is comparable. We found that the higher degree coefficients tend to have shorter periods than the lower degree coefficients (as shown in Figures A1 and A2).

### Forecasting core surface flow

**Fig. 6:**
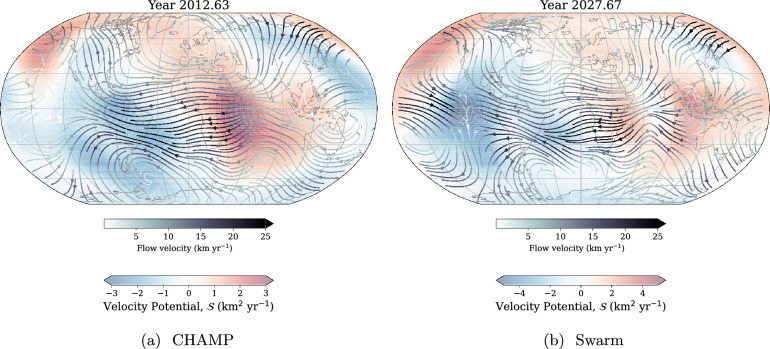
Snapshot of the predicted flow from the sinusoidal model from each satellite mission. Flow is superimposed on poloidal flow potential, $\mathcal {S}$, where $\mathcal {S}>0$ is indicative of fluid upwelling, and $\mathcal {S}<0$ is indicative of downwelling. Plot is in Robinson projection, and continents are shown for reference only

We obtain the forecast flow coefficients by first solving Eq. (11) for $\hat{\textbf{m}}_{t+1}$. This expression is then evaluated every 4 months, and extrapolated until 2015 and 2030 for CHAMP and Swarm, respectively. After obtaining flow coefficients from the discrete integration, we evaluate the core surface flow using Eqs. (4) and (5). Figure 6 shows a snapshot of the sinusoidal flow model at 2012.63 for CHAMP, and 2027.67 for Swarm, representative of our IGRF-11 and 14 models, respectively, around the midpoint of the forecasts obtained by integrating from the endpoints of the models (2010 and 2024 for CHAMP and Swarm, respectively). In both flows, the forecast retains features consistent with other studies, such as a persistent eccentric planetary gyre (e.g., Pais and Jault 2008), a strong westward jet underneath the Bering Strait (e.g., Livermore et al. 2017), and westward flow underneath the Atlantic ocean (e.g., Bullard and Gellman 1954). It is reassuring that the simplifications made in the prediction do not remove familiar flow features. Under the Pacific ocean, our sinusoidal model from CHAMP data manages to predict the overturn of the flow in the west-Pacific (as shown previously by, e.g., Kloss and Finlay 2019; Ropp and Lesur 2023; Finlay et al. 2023; Rogers et al. 2025; Grüne et al. 2025), but only in the region underneath the west Pacific. For Swarm, our sinusoidal model predicts the eastward flow in 2025 to strengthen over time. (The predicted and model flow coefficients are shown in Figures A3 and A4.)

The predicted sinusoidal flows are superimposed on the poloidal flow potential, $\mathcal {S}$, which shows the magnitude of up- and downwelling, where $\mathcal {S}>0$ indicates upwelling. Although the predicted flow is dominated by large-scale toroidal flow, primarily in the eccentric planetary gyre, it also shows interesting poloidal flow patterns, mostly centred around low latitudes. Most notable is the strong downwelling underneath the equatorial east Pacific, where the westward Atlantic flow and eastward Pacific flow collide. This was not established in 2012, and so does not appear in our flow forecast in Fig. 6a, but is clear from Fig. 6b. Our sinusoidal model from Swarm predicts that this will weaken and move southward over the next 5 years, and a region of upwelling underneath Southeast Asia strengthen over time. At high northern hemisphere latitudes, we also see strengthening up- and downwelling on either side of the core surface below the Bering Strait, which is linked to the further acceleration of the high-latitude jet.

**Fig. 7:**
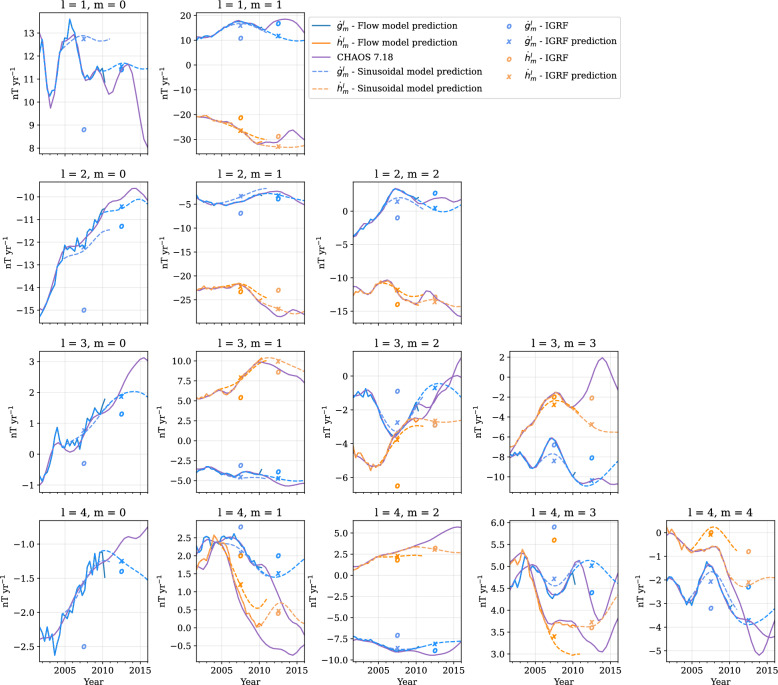
SV Gauss coefficients from 2001 to 2015 up to degree and order 4 for our CHAMP-based flows. Solid lines shows prediction from the flow model, and dashed lines show the candidate model predictions. Purple lines show CHAOS-7.18 values for reference (Finlay et al. 2020). Plusses mark the value of the SV coefficient reported for our candidate model, and open circles mark the values chosen for the IGRF-10 (2007.5) and IGRF-11 (2012.5). Vertical dotted black line marks the end time of the flow model. Note the change in *y*-axis scale for each subfigure

### Forecasting SV: comparing sinusoidal model from CHAMP to previous IGRFs

We now conclude the analysis of the CHAMP-based flows, by obtaining SV coefficients from the sinusoidal flow model, for every 4-month epoch, using Eq. (6). These are shown by dashed lines in Fig. 7, where SV coefficients from CHAOS$-$7.18 (purple lines, Finlay et al. 2020) and the core surface flow model predictions (solid lines) are also shown for reference. We see that, for most of the SV coefficients, our model is in good agreement with the core surface flow model and CHAOS for the period 2001.67–2010.00. We also see that after 2010, the forecast follows the CHAOS SV for most Gauss coefficients, for example, $\dot{h}_1^1$, $\dot{g}^2_0$, $\dot{g}_1^2$, and $\dot{h}_1^3$. There are some coefficients, however, whose time variation is more-complex than can be explained by a simple oscillation in core surface flow acceleration, notably $\dot{g}_1^1$ and $\dot{g}_3^3$, where we see a rapid in change in SV after 2010, which is not captured by our model. To evaluate our predictions in terms of IGRF-10 and 11, we obtain our IGRF candidate model coefficients by calculating the average value of each SV coefficient up to degree and order 8 in the time-period 2005–2010 and 2010–2015. Our final values are shown by the plusses in Fig. 7. Overall, our models are generally performing better during the period 2005–2010 than the IGRF-10, but less so for the period 2010–2015 than IGRF-11. However, the figure shows that our model is comparable to, or better than, IGRF-11 in predicting SV, with the exception of four coefficients; $\dot{g}_1^1$, $\dot{h}^2_2$, $\dot{h}_3^3$, and $\dot{g}_5^5$.

We compare our predictions, as well as previous IGRF predictions, to the observed geomagnetic field, as reported by the definite geomagnetic reference field (DGRF). We do this using the relevant DGRF as starting model, and summing the SV coefficients averaged over the next 5 years to predict the next DGRF. The root-mean-square (rms) difference in $\textbf{B}$ at the surface, between the predicted and actual DGRF is reported in Table 1. Our model has a quarter of the rms misfit for predicting the magnetic field in 2010, compared to that of IGRF-10. However, our model setup does a poorer job at predicting the magnetic field in 2015, where IGRF-11 has slightly lower rms error than our sinusoidal model.

**Fig. 8:**
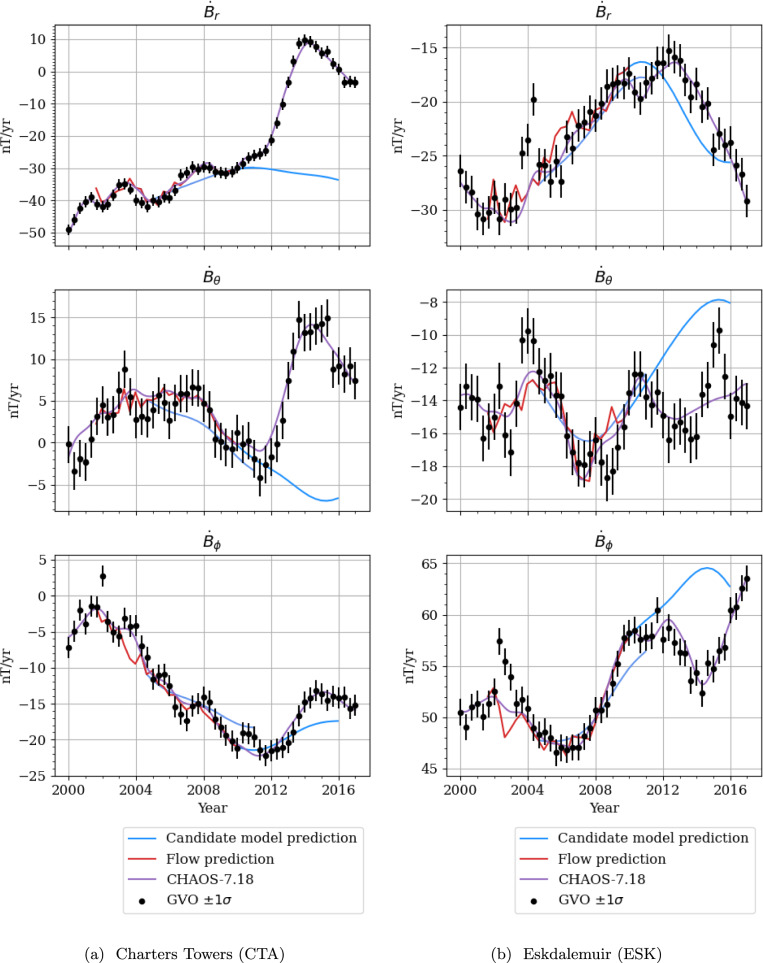
Vector SV at two ground observatories, one in eastern Australia (CTA) and one in United Kingdom (ESK). Red lines show SV predicted from the CHAMP flow model from 2001.63–2010.33, and blue points indicate forecasted SV from our candidate models. Note that the y-scale is different for each plot

Finally, we investigate the model’s ability to forecast the 2011 and 2014 geomagnetic jerks, by evaluating our SV prediction at two ground observatories, in which these jerks are clear: Charters Towers (CTA), Australia, and Eskdalemuir (ESK), United Kingdom. These are shown in Fig. 8. We see in this figure what we interpret as the poor forecast of the sectoral SV coefficients (as mentioned above), namely, the inability to capture the amplitude and polarity of the 2011 geomagnetic jerk. However, we note that the models were able to predict a geomagnetic jerk in 2014 (albeit of the wrong polarity in places,) as shown in Fig. 8b.

**Table 1:** RMS differences between predicted (from either previous iterations of IGRF or from our models based on flow acceleration) and the observed magnetic field at the Earth’s surface (using values from IGRF-14)

	DGRF-2010	DGRF-2015	DGRF-2020	IGRF-2025
IGRF-10–13 SV	$107.68~\text {nT}$	$79.68~\text {nT}$	$106.65~\text {nT}$	$103.5~\text {nT}$
Our models	$24.95~\text {nT}$	$83.23~\text {nT}$	$37.66~\text {nT}$	$38.1~\text {nT}$

**Fig. 9:**
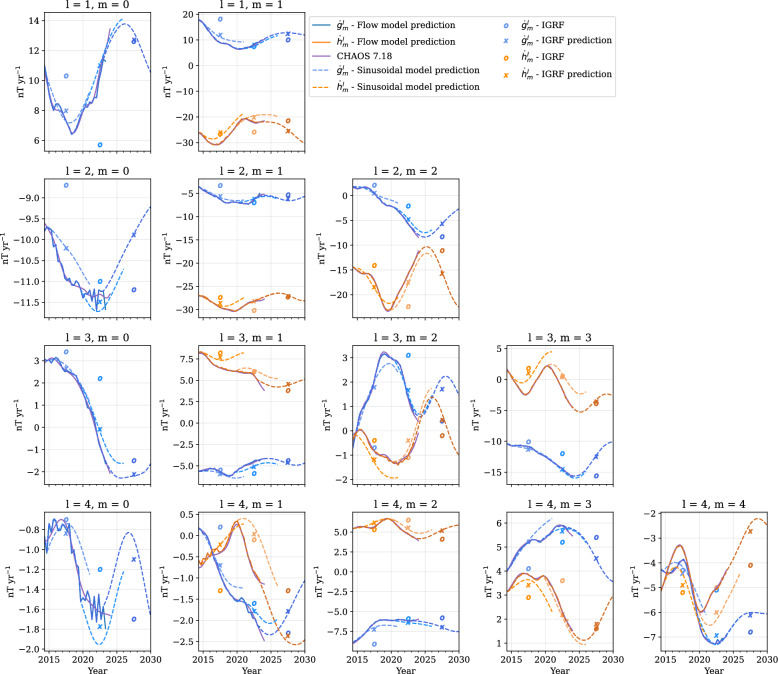
SV Gauss coefficients from 2014 to 2030 up to degree and order 4 for our Swarm-based flows. Solid lines shows prediction from the flow model, and dashed lines show the candidate model predictions. Purple dash–dotted lines show CHAOS-7.18 values for reference (Finlay et al. 2020). Crosses mark the value of the SV coefficient reported for our candidate model, and open circles mark the values chosen for the IGRF-12 (2017.5), IGRF-13 (2022.5) and IGRF-14 (2027.5). Note the change in *y*-axis scale for each subfigure

### Forecasting SV: evaluating our IGRF-14 candidate model

We now use our forecasts from the Swarm-based flows to obtain the candidate model for IGRF-14. Similar to above, we obtain SV coefficients from the sinusoidal flow model, every 4-month epoch, using Eq. (6), which are shown as dotted lines in Fig. 9. For most of the SV coefficients, our model is in good agreement with the core surface flow model and CHAOS for the period 2014.33–2024.00. We obtain our IGRF-14 candidate model coefficients by calculating the average value of each SV up to degree and order 8 in the time-period 2025–2030. Our final values are shown by the crosses in Fig. 9, and are provided in Supplementary Table A3. Note we do not estimate any formal uncertainties.

In Fig. 9, we also show the final IGRF-14 SV coefficients by the open circles. There is good agreement with some coefficients, for example, $(\dot{g},\dot{h})_4^4$ or $\dot{h}_3^0$. However, our values for other coefficients, for example, $(\dot{g},\dot{h})_3^3$, strongly deviate, and in the case of $\dot{h}_3^3$, represents the largest difference between our candidate model and the IGRF-14 of $5~{\mathrm{nT~yr}^{-1}}$. The rms misfit between our prediction and IGRF-14 is $0.84~{\mathrm{nT~yr}^{-1}}$. The misfit between our predictions and IGRF-14 are also presented in Supplementary Figure A5. Similar to the CHAMP-derived predictions, we report the rms error associated with our Swarm-based model’s ability to predicted the observed magnetic field at the surface in Table 1. Our model misfit is about a third that of the IGRF predictions for the 2020 DGRF and 2025 IGRF.

**Fig. 10:**
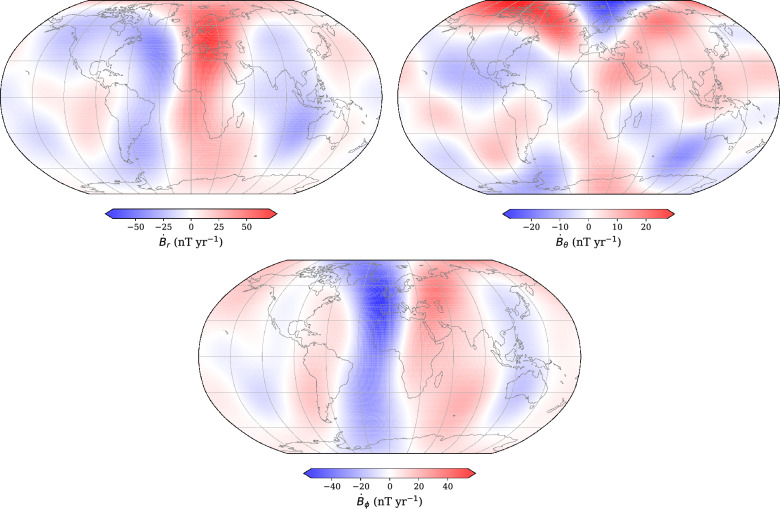
Radial ($\dot{B}_r$), meridional ($\dot{B}_\theta$), and azimuthal ($\dot{B}_\phi$) SV difference between our candidate model and the final IGRF-14 at 2027.5. The SV is plotted at the IGRF reference Earth radius ($a=6371.2$ km). Note that each plot has a different value range. Plots are in Robinson projection

We highlight the physical manifestation of the differences between our candidate model and the IGRF-14 in Fig. 10, where we have evaluated the differences in the SV components at the Earth’s surface. The most striking differences are in the *r* and $\phi$ components, with a band of high values centred on the Greenwich meridian, peaking beneath Europe, reminiscent of the signature of geomagnetic jerks as seen at the Earth’s surface (e.g., Finlay et al. 2020).

**Fig. 11:**
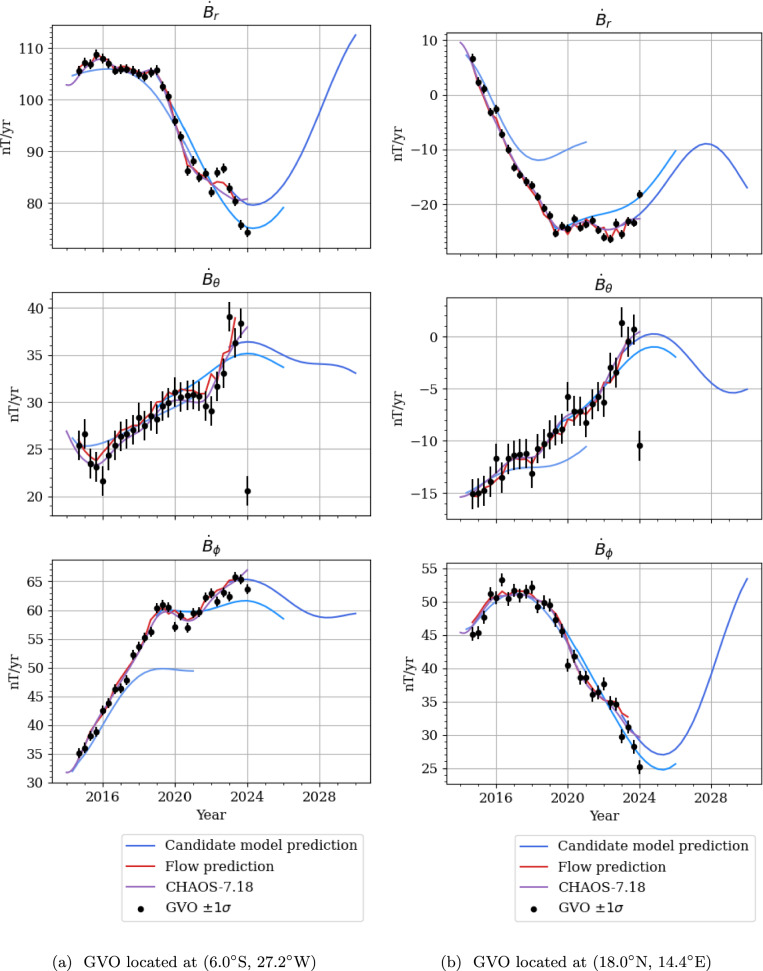
GVO vector SV at two low latitude GVOs, evaluated at Swarm altitude ($r=6861~\text {km}$). Red points are modelled from 2014.33 to 2024.00, and blue points indicate forecasted SV until 2030. Note that the y-scale is different for each plot

To investigate jerk-like signatures, we predict the SV vector from our candidate model, as it would be measured at two low latitude Swarm GVOs; one in the equatorial Atlantic (Fig. 11a), and one in central Africa (Fig. 11b). These are compared to SV vector data from Swarm, the SV generated by advection from the flow model, and CHAOS-7.18. Our candidate model matches the overall trends of the observed SV at these two locations, as compared to the Swarm data and CHAOS$-$7.18 for the time period 2014.33–2024. In particular, we see that our model forecast from 2015 to 2020 was able to capture the 2017 jerk. Figure 11 also shows how the candidate model forecasts inflections in SV—which we associate with geomagnetic jerks—in 2024 and 2028. The 2024 jerk is clearest in the radial component in Fig. 11a, whereas the 2028 jerk only occurs in the radial component in Fig. 11b.

## Discussion

Our recreation of earlier IGRF candidate models suggests that this method of forecasting SV over the following 5 years largely outperforms the average IGRF predictions in the past. The exception is for the period 2010–2015, where our forecast had strong deviations from the real SV variation, which we attribute to large-scale changes in the core environment around 2010, rather than an inherent flaw in our modelling strategy. Most directly related is the overturn of the core surface flow underneath the Pacific (e.g., Kloss and Finlay 2019; Ropp and Lesur 2023), during which Grüne et al. (2025) reported a redistribution of power in the toroidal and poloidal flow power spectrum, and the concentration of two localised patches of poloidal flow—indicative of up- and downwelling—either side of the Pacific. This time also marks the onset of waves of a period of 3.5 years beneath the Pacific (Gillet et al. 2024). Beyond studies of core surface flow and magnetic field, Madsen and Holme (2025) have reported on the interruption of the dominant 6-year oscillation in length-of-day in 2010, returning in amplitude (but changed in phase) in 2014. (This is similarly reported, but not commented on, by Saraswati et al. (2023) and Sidorov et al. (2025) when looking at period changes in the magnetic field.) This 2010 interruption in the 6-year oscillation is also contemporary with seismological evidence for change in inner-core seismic signature (Yang and Song 2023; Wang et al. 2024; Vidale et al. 2025). Madsen and Holme (2025) argue that this could have been initiated by the release of less dense material at the inner core boundary, resulting in outward propagation of lighter elements, and thus triggering magnetic waves which propagate through the outer core (e.g., Aubert and Gillet 2021; Aubert et al. 2022; Finlay et al. 2023). This train of events could be the main cause for these observed changes, such as the disruption of the 6-year oscillation in length-of-day (Madsen and Holme 2025) and the changed flow underneath the Pacific (e.g., Kloss and Finlay 2019; Ropp and Lesur 2023; Grüne et al. 2025). It is, therefore, likely that the disruption to clear periodic features of the core (e.g., Holme and de Viron 2013; Gillet et al. 2024), all occurring around 2010, is the main reason for our model’s deviation from observations. In this case we can remain confident in our predictions for 2024–2030, based on the Swarm-derived flows, assuming that we will not see a major overturn in large-scale flow over the next 5 years.

Figure 9 shows our candidate model is in good agreement with the SV generated by the input flow model and CHAOS$-$7.18 for the majority of the SV coefficients in the Swarm era. Although there is no direct link between variations in SV spherical harmonic coefficients and geomagnetic jerks, our sinusoidal model allows for non-linear variations, permitting the existence of geomagnetic jerks (e.g., Brown et al. 2013; Aubert et al. 2022), otherwise not captured by linear forecast methods.

**Fig. 12:**
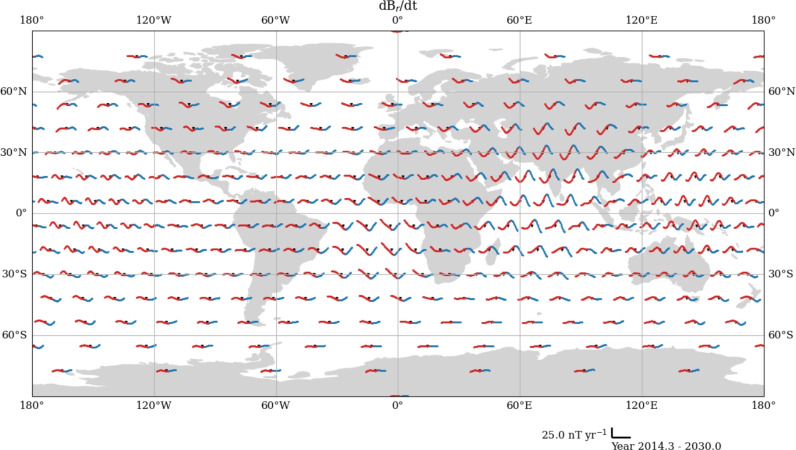
Predicted radial SV from our candidate model at 300 GVO locations, evaluated at Swarm altitude ($r=6861~\textrm{km}$). Red points are evaluated from 2014.33 to 2024.00, and the blue are the forecast until 2030. Scale-bar is given in the bottom right. The figure is plotted using Plate Carree projection

Figure 12 shows the radial secular variation at the 300 GVO locations used to represent Swarm data (e.g., Hammer et al. 2021, 2022). In red, we show the SV modelled during the period of available data, and in blue, we show the predicted SV from our model. Our model was able to capture the 2017 (Kloss and Finlay 2019; Finlay et al. 2020; Whaler et al. 2022) and 2020 (e.g., Pavón-Carrasco et al. 2021; Madsen et al. 2025) jerks. Furthermore, we see that our candidate model predicts a geomagnetic jerk in 2024, most apparent in the western Atlantic and the western Pacific. Madsen et al. (2025) associate jerks in 2017 and 2020 with the times of the beginning and end of a localised pulse in azimuthal core surface flow acceleration, and predicted a geomagnetic jerk in the equatorial Pacific arising from the collapse of another such pulse in 2024. Our sinusoidal model, which is an extrapolation of their flow model, makes the same prediction, but also suggests the occurrence of a geomagnetic jerk in the Atlantic, contemporaneous with the Pacific 2024 jerk. Furthermore, Fig. 12 forecasts a geomagnetic jerk occurring in 2028 in central Africa, as also shown in Fig. 11b. These two jerks may be associated with a pulse in modelled surface-flow acceleration underneath equatorial east Africa between 2024 and 2028, similar to that observed by Madsen et al. (2025) in the equatorial Pacific between 2018 and 2022, as they occur at times of low azimuthal acceleration (see Supplementary Figure A6).

As noted in Sect. 4, our flow model predicts a weakening of the downwelling beneath the east equatorial Pacific. Ropp and Lesur (2023) first noted a change in flow direction beneath the Pacific from westward to eastward (although Grüne et al. 2025, were the first to pinpoint the time of this direction reversal to 2011); our sinusoidal model predicts a strengthening of this flow throughout the 2020s. In comparison with beneath the Pacific, our model predicts relatively little change underneath the Atlantic. This is in contrast to the 2000s, where the flow underneath the Pacific was quasi-steady, and the Atlantic region saw more dynamic variation (as noted by, e.g., Chulliat and Maus 2014, investigating secular acceleration).

Our IGRF-14 candidate model is based on assumed periodic dynamics of core surface flow. This is primarily inspired by the growing evidence for hydromagnetic waves propagating along the CMB (Gillet et al. 2022; Istas et al. 2023; Finlay et al. 2023; Gerick and Livermore 2024). With the increased data quality from satellite measurements, as well as quasi-continuous near-global sampling of the geomagnetic field from satellites over the past 26 years, we are now able to investigate intradecadal periodic variations in core surface flow. As shown in Fig. 3, we can reasonably fit individual time-varying flow-acceleration coefficients over the past 10 years with a single period sinusoid. Although this vastly simplifies the complex magnetohydrodynamics that interplay in the core, our approach offers a dynamic prediction that does not asymptote to infinite flow velocity over time, unlike a polynomial fit, for example.

## Conclusions

We present a predictive model for SV calculated from a model of periodically varying flow acceleration at the core surface. The flow velocity is given by the integral of this function, from an initial value, or equivalently an arbitrary constant—in our case, the values for the first epoch of our initial flow model. Such a near-constant flow (on a decadal timescale) yields an SV model broadly consistent with those obtained by conventional prediction, most specifically within the framework of the IGRF. Our flow-predicted SV suggests geomagnetic jerks occurring in the Pacific in 2024 (as has also been suggested by Madsen et al. 2025; Madsen and Holme 2025) and in central Africa in 2028. Adopting an oscillatory model for variations in this flow has the additional benefit that if extended outside the timespan of the prediction it will remain bounded, which is not the case for some other possible approaches, such as polynomial or exponential temporal variations. Oscillatory variations are consistent with the idea that intra- to bidecadal core surface dynamics are dominated by periodic behaviour (e.g., Gillet et al. 2022). This model was incorporated into the IGRF-14 secular variation prediction.

## Supplementary Information

Supplementary file 1

## Data Availability

The Swarm SV spatial gradient GVO data used in our model are available from JBL. The CHAOS-7.18 model coefficients of Finlay et al. (2020) were obtained from https://www.spacecenter.dk/files/magnetic-models/CHAOS-7/ [Last accessed 26/11/2024], and the COV-OBSx2 model coefficients of Huder et al. (2020) were obtained from http://www.spacecenter.dk/files/magnetic-models/COV-OBSx2/ [Last accessed 13/08/2025]. The IGRF-14 coefficients are available from Zenodo with the index: (doi:10.5281/zenodo.14205635). The code developed for our models is available through GitHub: https://git.ecdf.ed.ac.uk/s1755034/FDMadsen_PhD.

## Abbreviations

CMB: Core–mantle boundary
GVO: Geomagnetic virtual observatory
IGRF: International geomagnetic reference field
SV: Secular variation
rms: Root-mean square
DGRF: Definite geomagnetic reference field
