# Supplementary material for: IGRF-14 secular variation prediction from core surface flow acceleration
Source: Earth Planets Space. 2026 Jan 16;78(1):29. doi: 10.1186/s40623-025-02347-x (PMC12891054; doi:10.1186/s40623-025-02347-x)
Supplement: Supplementary file 1 [file 40623_2025_2347_MOESM1_ESM.pdf]

Table A2. : Spatial and temporal damping coefficients, λ_v and λ_t, and normalised rms misfit for the flows using gradient tensor from CHAMP and Swarm, as presented in Madsen et al. (2025). The spatial norm value is calculated from Equation (9.)

		CHAMP	Swarm
Spatial damping	λ_v	10^{-3}	6×10^{-4}
Temporal damping	λ_t	10^3	10^3
Normalised rms misfit		1.08	1.08
Spatial norm value ($\mathrm{km}^{-2}\,\mathrm{yr}^{-2}$)		10.99×10^6	25.73×10^6

Appendix A. Supplementary material

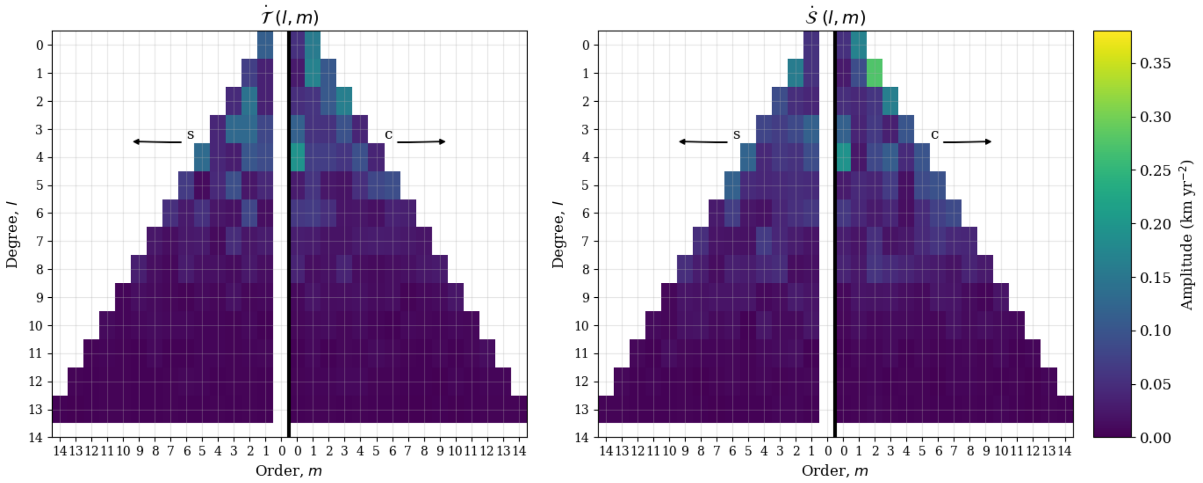

(a) CHAMP – amplitude

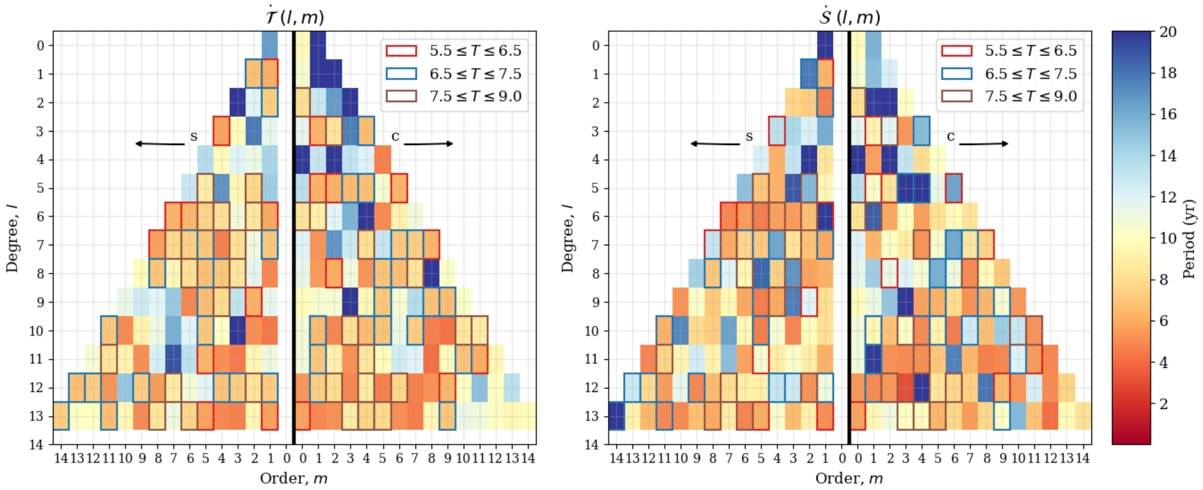

(b) CHAMP – period

Figure A1. : Amplitude (a) and period (b) of sinusoidal fits to each toroidal (left) and poloidal (right) flow acceleration coefficient from the CHAMP-derived flow. $t_l^{m\,s}$ and $s_l^{m\,s}$ are on the left side, and $t_l^{m\,c}$ and $s_l^{m\,c}$ are on the right side of the plots, for toroidal and poloidal flow-acceleration coefficients, respectively. Periods in the vicinity of 6 (red), 7 (blue), and 8.5 years (brown) have been highlighted.

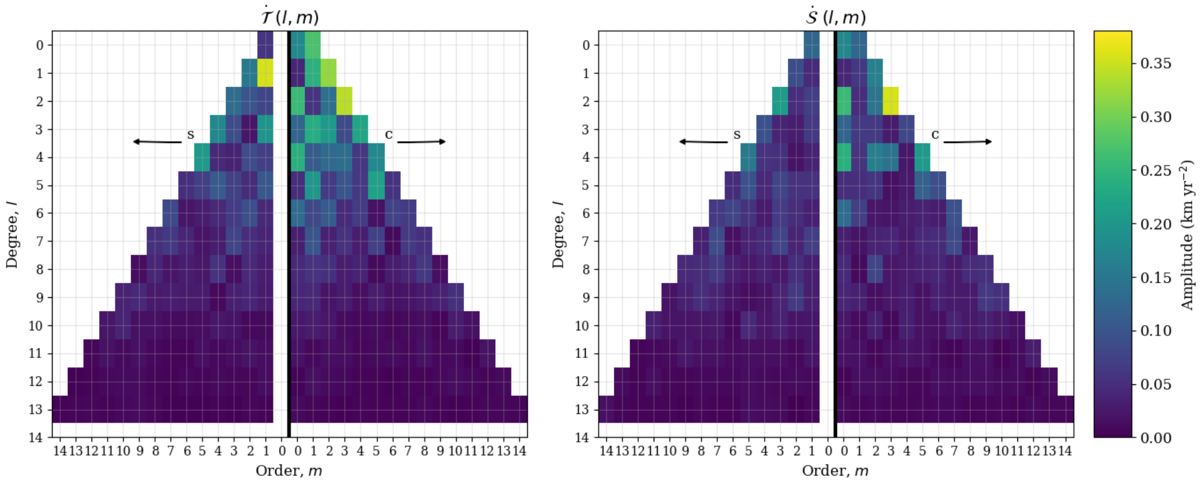

(a) Swarm – amplitude

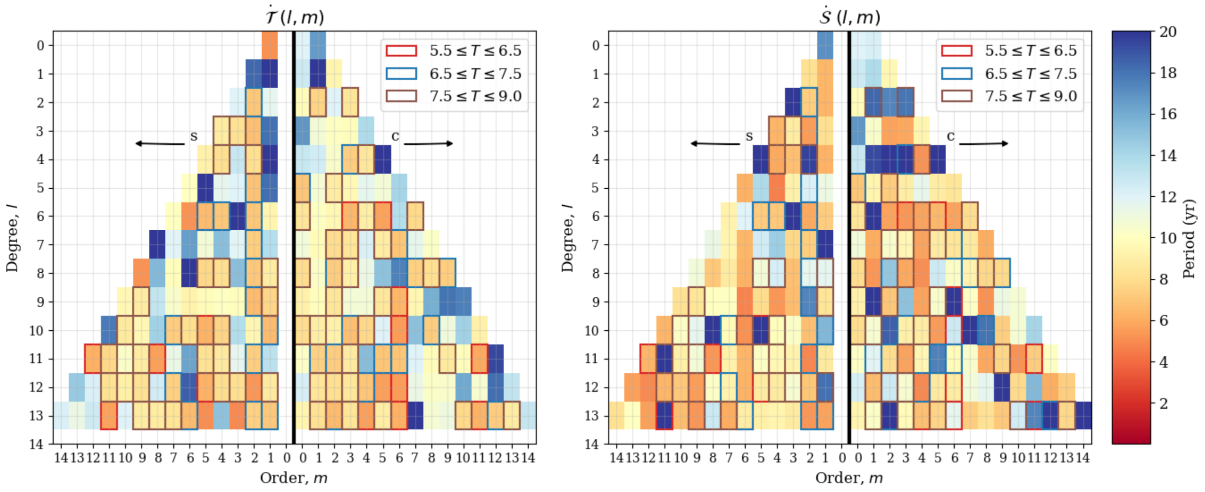

(b) Swarm – period

Figure A2. : Same as Figure A1, but for Swarm.

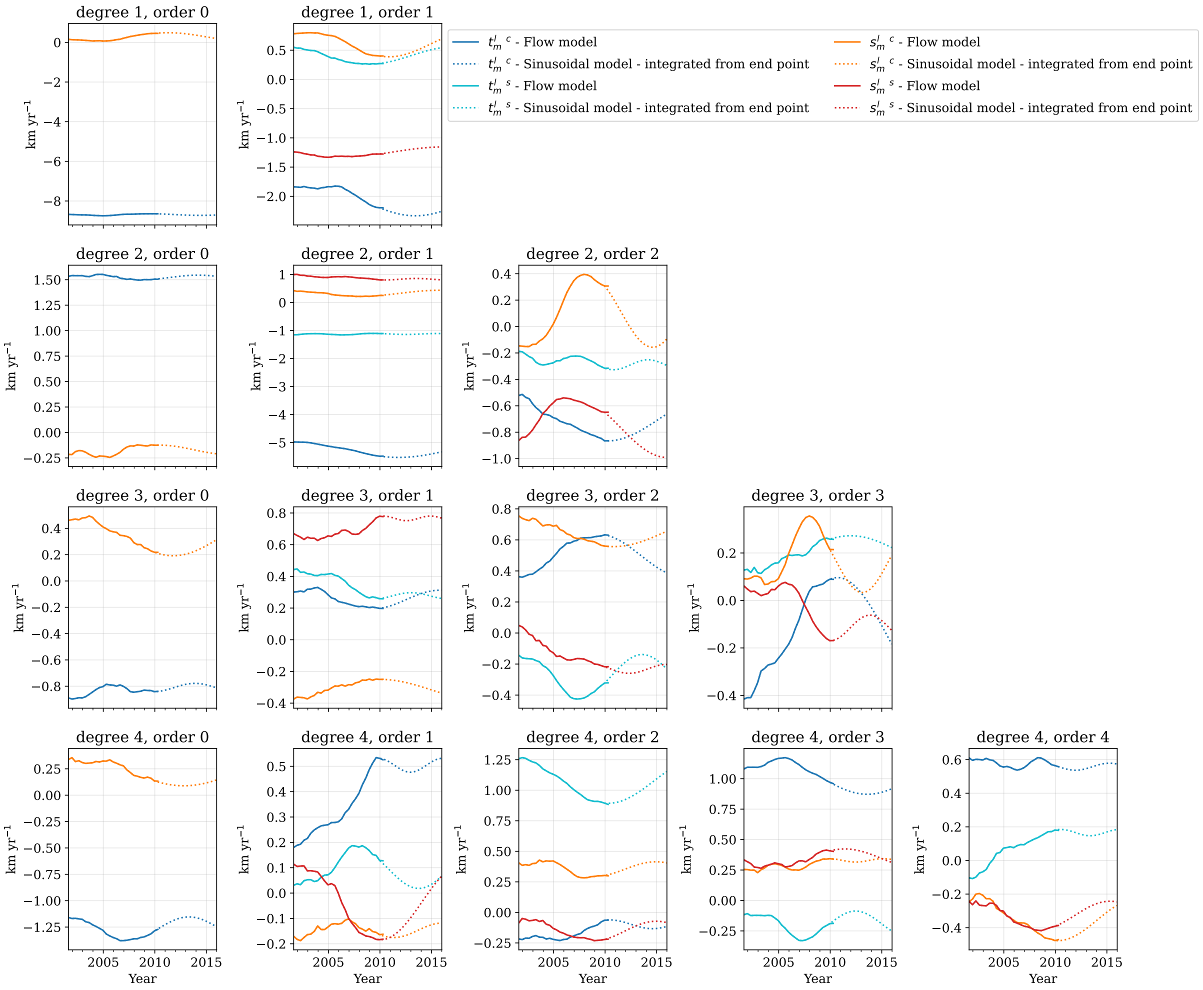

Figure A3. : Toroidal (blue and turquoise) and poloidal (orange and red) flow coefficients up to degree and order 4 for CHAMP. Solid lines show flow from the core surface flow model (Madsen et al. 2025), and dotted lines show prediction from sinusoidal fit to acceleration. Rows show spherical harmonic degree, and columns spherical harmonic order. Note that the y-axis scale varies for each order and degree.

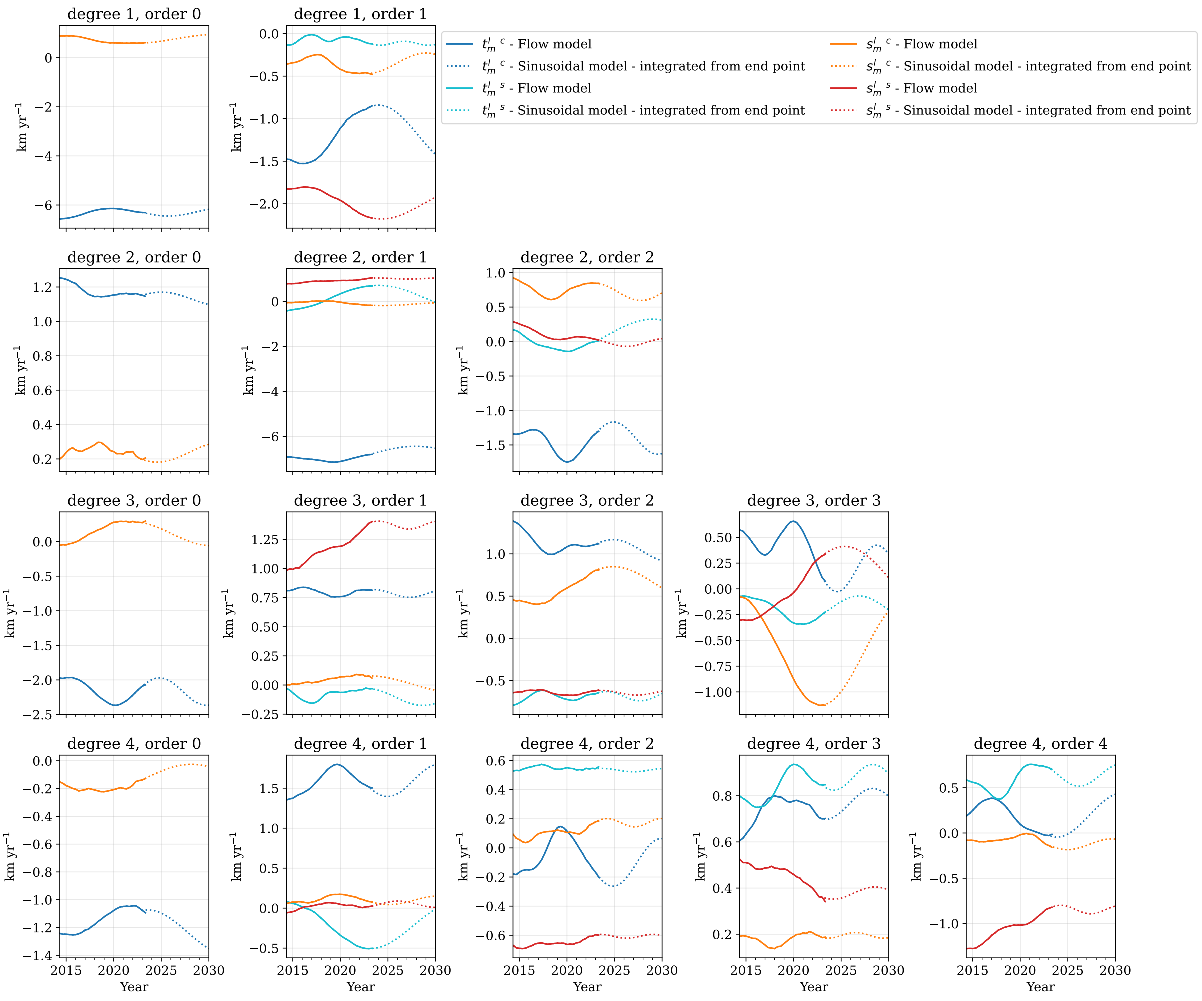

Figure A4. : Same as Figure A3, but for Swarm.

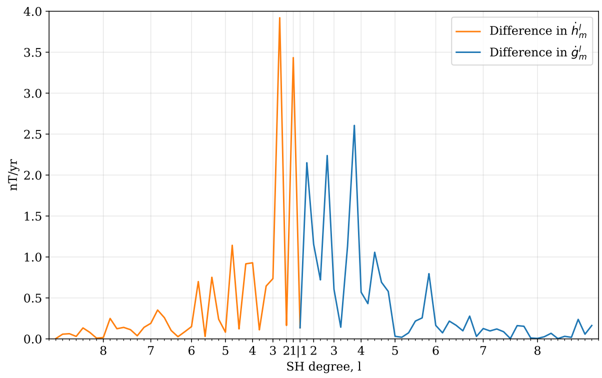

Figure A5. : Difference between our candidate model and the final IGRF-14 as a function of spherical harmonic degree. Note that x-axis should be read from right-to-left for the section related to $\dot{h}^l_m$.

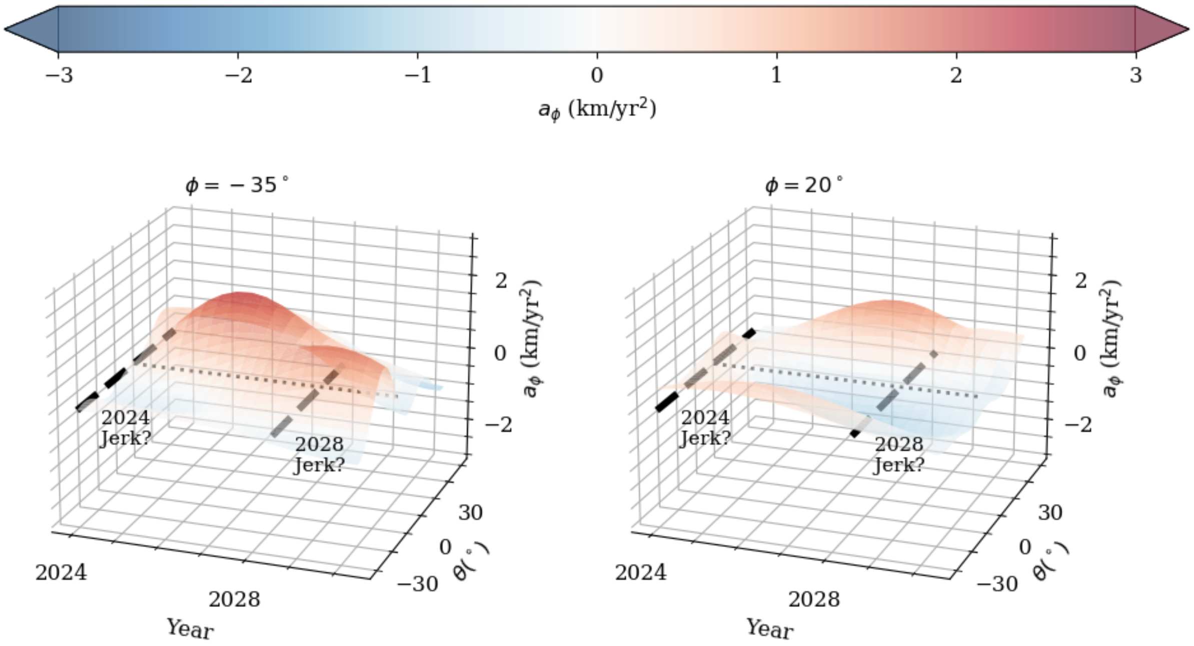

Figure A6. : Time-latitude plots of azimuthal flow acceleration, a_ϕ, from the candidate model, covering $\pm 30^\circ$ latitude (where θ is latitude, rather than colatitude, in this figure only) at constant longitudes (ϕ) of -35° (left) and 20° (right). $a_\phi > 0$ signifies eastward acceleration, and $a_\phi < 0$ signifies westward acceleration.

Table A3. : IGRF-14 candidate SV coefficients for 2025–2030 in units of nT yr^{-1}.

l	m	$\dot{g}_l^m$	$\dot{h}_l^m$
1	0	13.34	—
1	1	13.97	-20.79
2	0	-9.84	—
2	1	-5.89	-25.40
2	2	-3.16	-16.12
3	0	-1.27	—
3	1	-5.89	7.34
3	2	1.55	-0.29
3	3	-12.40	1.42
4	0	-0.78	—
4	1	-1.10	-1.42
4	2	-8.06	5.82
4	3	5.08	0.99
4	4	-5.19	-4.40
5	0	0.21	—
5	1	0.64	-0.34
5	2	-0.79	1.80
5	3	0.20	-1.93
5	4	2.16	3.62
5	5	2.41	1.44
6	0	-0.64	—
6	1	0.01	0.17
6	2	0.27	-1.94
6	3	1.27	-1.18
6	4	-1.20	0.55
6	5	0.18	1.56
6	6	1.18	0.41
7	0	0.17	—
7	1	-0.45	0.81
7	2	-0.10	0.47
7	3	0.89	-0.44
7	4	0.26	-0.24
7	5	-0.67	-0.90
7	6	-0.94	0.30
7	7	0.69	-0.24
8	0	-0.02	—
8	1	0.27	-0.23
8	2	-0.27	0.35
8	3	0.46	0.01
8	4	-0.02	0.65
8	5	0.28	-0.30
8	6	0.27	-0.25
8	7	-0.12	0.45
8	8	0.05	0.18
